# Factors Influencing the Total Functional Capacity Score as a Critical Endpoint in Huntington’s Disease Research

**DOI:** 10.3390/biomedicines11123336

**Published:** 2023-12-17

**Authors:** Jannis Achenbach, Benjamin Stodt, Carsten Saft

**Affiliations:** 1 Department of Neurology, Huntington Center North Rhine-Westphalia, St. Josef-Hospital Bochum, Ruhr-University Bochum, Gudrunstraße 56, 44791 Bochum, Germany; carsten.saft@rub.de; 2 Leibniz Research Center for Working Environment and Human Factors at the Technical University of Dortmund (IfADo), Ardeystraße 67, 44139 Dortmund, Germany; stodt@ifado.de

**Keywords:** Huntington’s disease, Total Functional Capacity (TFC), disease stages, neuropsychiatry, pre-manifest HD, moderated regression analysis, ENROLL-HD

## Abstract

**Background**: The Total Functional Capacity (TFC) score is commonly used in Huntington’s disease (HD) research. The classification separates each disease stage (1–5), e.g., as an inclusion criterion or endpoint in clinical trials accepted by the Food and Drug Administration (FDA). In addition to the quantification of age- and CAG-repeat-dependent effects as well as interacting effects of both on the TFC, we aimed to investigate factors influencing the TFC, such as neuropsychiatric, educational, and cognitive disease burden using data from the largest HD observational study to date. In addition, we analyzed data from pre-manifest stages to investigate the influence of the above-mentioned factors on the TFC in that stage. **Methods:** A moderated regression analysis was conducted to analyze the interaction effects of age and CAG-repeat length on the TFC in HD patients. A simple slope analysis was calculated to illustrate the effects. Depending on TFC results, motor-manifest patients were grouped into five stages. Data from pre-manifest participants were analyzed with regard to years to onset and CAP scores. **Results:** We identified *N* = 10,314 participants as manifest HD. A significant part of variance on the TFC was explained by age (*R*^2^ = 0.029, *F* (1;10,281) = 308.02, *p* < 0.001), CAG-repeat length (∆*R*^2^ = 0.132, ∆*F* (1;10,280) = 1611.22, *p* < 0.001), and their interaction (∆*R*^2^ = 0.049, ∆*F* (1;10,279) = 634.12, *p* < 0.001). The model explained altogether 20.9% of the TFC score’s variance (*F* = 907.60, *p* < 0.001). Variance of psychiatric and cognitive symptoms significantly differed between stages. Exploratory analysis of median data in pre-manifest participants revealed the highest scores for neuropsychiatric changes between 5 to <20 years from the disease onset. **Conclusions:** TFC is mainly explained by the neurobiological factors, CAG-repeat length, and age, with subjects having more CAG-repeats showing a faster decline in function. Our study confirms TFC as a robust measure of progression in manifest HD.

## 1. Introduction

The fatal autosomal-dominant inherited Huntington’s Disease (HD) is accompanied by manifold motor, cognitive, behavioral-psychiatric, and functional impairments [1,2,3]. To objectify psychiatric symptoms and disease-specific functional impairments, various research approaches have been followed to gain insights into underlying genetic and pathophysiological changes [4,5,6,7,8]. The distinct cause of disease with a cytosine-adenine-guanin (CAG)-trinucleotide expansion on chromosome 4 in the Huntingtin gene (HTT) results in misfolded Huntingtin proteins. Longer CAGs result in an earlier age of the motor onset (AAO) [1,9]. But the psychiatric onset of the disease, too, is partly associated with CAG-repeat length as revealed by Vassos et al. and McAllister et al [10,11]. Research suggests that the expanded CAG length explains about 50% to 70% of the variance in the AAO [12,13,14]. Langbehn et al. developed predicting models based on repeat length and validated findings in large real-world cohorts [15,16]. CAG-length-dependent effects negatively influencing morphological changes and correlates of the course of disease were investigated using different approaches in smaller cohorts [17,18,19,20]. With regard to neuropathological changes investigated in brain tissues with striatal atrophy, Penney et al. identified correlations between pathologic CAG-polyglutamine lengths and the age of affected HD patients, postulating linear pathologic changes from birth [21]. To quantify an index of the cumulative toxicity of the genetic burden and age, the CAG-age product (CAP)-score was developed as a data-driven approach [22].

The main focus has been on function and neuropsychological (cognitive) disabilities in HD across different stages and the longitudinal disease manifestation [23,24]. To assess motor, cognitive, neuropsychiatric, and functional symptoms, helpful rating scales are validated for HD using the Unified Huntington’s Disease Rating Scale (UHDRS) [4,25]. As part of the UHDRS, the Total Functional Capacity (TFC) score is used to evaluate functional impairments in the domains of occupation, finance, domestic chores, daily living activities, and care level. Depending on the TFC, motor-manifest participants are grouped according to stage 1 (TFC Score 131), 2 (TFC Score 10-7), 3 (TFC Score 6-3), 4 (TFC Score 2-1), and 5 (TFC Score 0). These disease stages are relevant, for example, as inclusion criteria for clinical trials, and the TFC score is investigated as a relevant endpoint.

However, earlier research showed that cognitive impairments and different motor phenotypes may also have an influence on functional capacities in manifest HD [26,27,28]. In particular, dystonia and hypokinetic rigidity have a stronger influence on functional impairments than chorea [2,27]. After reviewing 14 PubMed-published articles, Sellers et al. found some evidence for depression and apathy being associated with decreased functional capacities in Huntington’s disease [29]. As a conflicting result, the influence of cognitive burden and neuropsychiatric symptoms was not confirmed by a study by Gibson et al. in early-stage HD [30]. A more recently published review concluded that further investigations are necessary to gain a better understanding of the mechanisms caused by CAG-repeat-dependent changes occurring alongside different HD phenotypes and symptoms [31]. Neuropsychiatric symptoms have a high prevalence of 33–76% for depression, anxiety, irritability, and apathy in HD [32,33]. The influence of these symptoms on disease progression and, especially, on functional impairments in pre-manifest stages of the disease, however, remains unclear.

In the following sections, we set out to investigate the ENROLL-HD database with regard to the severity of neuropsychiatric, cognitive, and stage-dependent impairments in motor- and pre-manifest HD participants with different functional impairments.

## 2. Methods

### 2.1. ENROLL-HD Database with Regard to Functional Classification

ENROLL-HD is a global clinical research platform designed to facilitate clinical research in HD [34]. Core datasets are collected annually from all research participants as part of this global multicenter longitudinal observational study. Data are monitored for quality and accuracy using a risk-based monitoring approach. All sites are required to obtain and maintain local ethics approval. We investigated the periodic dataset 5 (PDS5) and identified 21,116 individual participants [35,36]. The analyzed data were collected as part of the global clinical research platform with participants from North America, Europe, Australasia, and Latin America, starting in 2012 and actively recruiting. The assessed periodic dataset 5 was created before the year 2020.

Ethics approval was obtained from the local ethics committee of Ruhr-University Bochum (No. 4941-14).

To compare disease stages of HD patients with symptoms of different severity with regard to individual functional impairments, we analyzed TFC subscales of the UHDRS functional assessment as defined for stages 1–5 [37,38]. Pre-manifest participants were grouped into five groups according to the predicted onset of <5, 5 to <10, 10 to <15, 15 to <20, and >20 years (with a desired onset probability of 0.6) using the onset calculator developed by Langbehn et al [15].

As inclusion criteria for motor-manifest participants, we set age (≥18 years), a diagnostic confidence level (DCL) of 4 (having unequivocal signs of clinical manifest HD: >99% confidence), a total motor score (TMS) ≥ 5, and a genetically confirmed report with ≥36 cytosine-adenine-guanine (CAG) repeats in the Huntingtin gene (HTT), resulting in a total sample of *n* = 10,314 participants. As fundamental demographic and genetic parameters, we analyzed age, CAG-repeat length, sex, disease duration, HD diagnosis, motor onset, and total motor score (UHDRS). We additionally calculated CAP scores [22].

With regard to neuropsychiatric symptoms, we investigated the standardized Problem Behaviours Assessment-short (PBA-s) questionnaire, as reported by the clinical rater, analyzing sub-scores for depression, irritability/aggression, psychosis, apathy, and executive functions, as well as self-reported assessments using the Hospital Anxiety and Depression Scale/Snaith Irritability Scale (HADS-SIS) with sub-scores for anxiety, depression, irritability, and outward/inward irritability implemented within the ENROLL-HD clinical visits. Neuropsychiatric sub-scores were calculated based on the frequency and severity of observed symptoms. In terms of cognitive symptoms, we compared performances in the Symbol Digit Modalities, verbal fluency (category), Stroop color naming, Stroop word reading, and Stroop interference test.

### 2.2. Statistical Analysis

Differences between TFC subgroups were analyzed using multigroup ANOVA analyses. Subsequently, post hoc Tukey HSD was performed to analyze pairwise differences with regard to neuropsychiatric symptoms between disease stages and pre-manifest participants according to calculated years to onset. Chi-square tests were used for analyses of categorical variables. Further, we calculated a moderated regression analysis to identify the influence of age, CAG-repeat length, and their interaction on the functional capacity of motor-manifest HD patients. A simple slope analysis was calculated to illustrate the identified interaction effects. All analyses were calculated using IBM SPSS Statistics V.28.

## 3. Results

### 3.1. Motor-Manifest Participants from ENROLL-HD According to Individual Disease Stages

**Figure 1:**
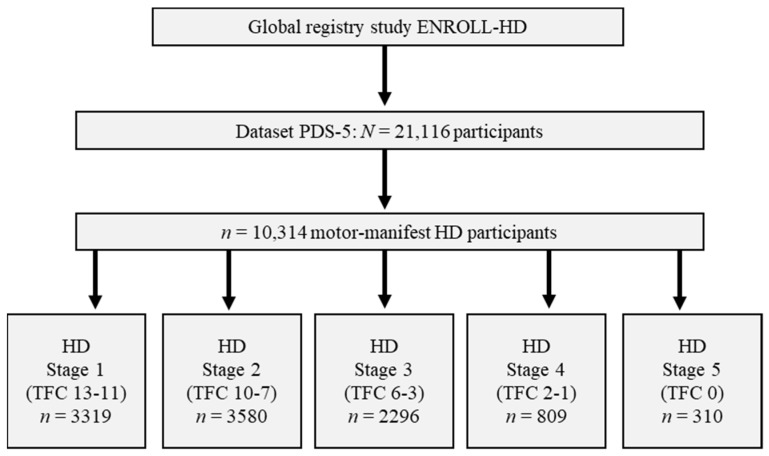
Data analysis of motor-manifest participants from ENROLL-HD according to disease stages. Abbreviations: PDS-5: Periodic dataset 5; HD: Huntington´s Disease; TFC: Total Functional Capacity.

After analyzing the functional status (TFC) of all participants matching the aforementioned criteria, *n* = 3319 appeared to be classified as stage 1, *n* = 3580 as stage 2, *n* = 2296 as stage 3, *n* = 809 as stage 4, and *n* = 310 as stage 5, respectively (Figure 1).

We found significant group differences in demographic, genetic, onset, and motor parameters between disease stages of HD participants affected to varying degrees (all *p* < 0.001). Additionally, the calculated CAP scores showed age-adjusted genetic differences between stages (*p* < 0.001; Table 1).

### 3.2. Neuropsychiatric Symptoms According to Different Disease Stages

Neuropsychiatric parameters were analyzed within the PBA-s as the assessment of the clinical rater and within the HADS-SIS as self-reported psychiatric burdens. Significant group differences within baseline neuropsychiatric data revealed mean group differences for all analyzed sub-domain scores within the PBA-s (all *p* < 0.001) and the HADS-SIS (all *p* < 0.050).

Highest group domain scores within the PBA for depression were observed in stages 2 and 3, whereby sub-domains for irritability/aggression, psychosis, apathy, and executive functions revealed higher mean sub-scores (more impairment) in disease stages with more functional impairments (stages 4 and 5). Remarkably, self-reported neuropsychiatric impairments in the HADS-SIS scored highest for depression in stages 4 and 5 as well as for irritability, outward and inward irritability in stages 2 and 3 (Table 2).

The subsequent post hoc analyses showed higher group domain scores for depression (PBA) in stage 2 and 3 patients than in stage 1 patients (all *p* < 0.001). No significant differences were observed for the PBA-depression sub-score in all other group comparisons. Further pairwise comparisons within the domains of irritability/aggression, apathy, and executive functions indicated that groups suffering from higher functional impairments had more impairments within most neuropsychiatric sub-scores. Less pronounced differences between stages were observed within the HADS-SIS anxiety sub-score, with no significant differences between stages, except for the comparison of stage 1 and higher sub-scores in stage 2. The sub-score for depression indicated more impairments in stages with more functional impairments. Irritability and outward irritability revealed—similar to the anxiety sub-score—no differences in post hoc comparisons except for stage 1 vs. stage 2 with more impairments in the latter (Table 3).

### 3.3. Interaction Effect between Age and CAG-Repeat Length in Motor-Manifest HD

**Figure 2:**
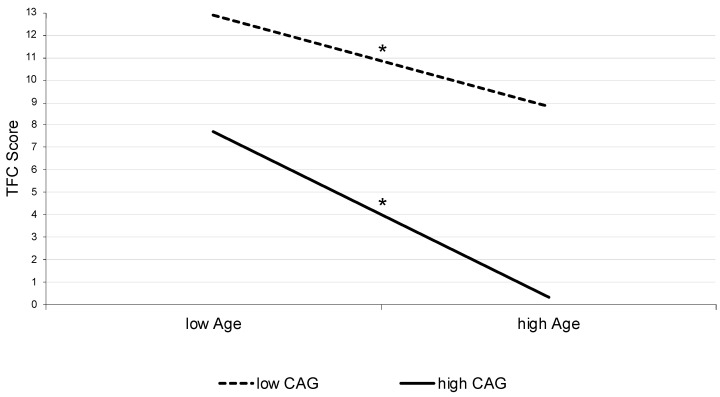
Interaction effect illustrated as simple slopes. Abbreviations: CAG: cytosine-adenine-guanine repeat length; TFC: Total Functional Capacity. * *p* < 0.001.

In addition to the comparison of different functional impairment stages with special regard to neuropsychiatric symptoms, we calculated a moderated regression analysis to identify the influence of age and CAG-repeat length on the functional capacity of HD patients with motor manifestations and to illustrate possible interaction effects. Here, we identified that age accounted for a significant amount of variance in the TFC score (*R*^2^ = 0.029, *F* (1;10,281) = 308.02, *p* < 0.001). Furthermore, CAG-repeat length (∆*R*^2^ = 0.132, ∆*F* (1;10,280) = 1611.22, *p* < 0.001), and the interaction of both variables explained an additional significant part of variance (∆*R*^2^ = 0.049, ∆*F* (1;10,279) = 634.12, *p* < 0.001), resulting in an overall explanation of altogether 20.9% within the TFC score´s variance (*F* (3;10,278) = 907.60, *p* < 0.001). Additionally, a simple slope analysis was calculated to illustrate the identified interaction effect. Both slopes, for low CAG score (*b* = −0.16, *t* = 36.97, *p* < 0.001) as well as for high CAG score (*b* = −0.29, *t* = 49.32, *p* < 0.001), were significantly different from zero (Figure 2).

### 3.4. Neuropsychiatric Symptoms in Pre-Manifest HD According to Calculated Years to Onset

Further, we compared neuropsychiatric symptoms in pre-manifest HD participants according to predicted years to onset [15]. Out of *n* = 5149 pre-manifest HD participants, we identified *n* = 138 with a predicted onset of <5 years; *n* = 1242 predicted 5–<10; *n* = 1215, 10–<15; *n* = 920, 15–<20 and *n* = 1634 predicted >20 years to onset (Table 4).

Group comparisons and post hoc analysis regarding the PBA-s in pre-manifest participants revealed increased tendencies toward depressive symptoms in those with a predicted onset between 5 to <10 years and 10 to <15 years compared with participants with a predicted onset further than 20 years. The same differences were found for apathy (all *p* < 0.010). No distinct group differences were observed for irritability, psychosis, and executive functions. Analysis of the HADS-SIS sub-scores revealed no differences between stages with regard to anxiety. Participants with 5–<10, 10–<15, and 15–<20 years to onset revealed significantly more depression than those with a predicted onset >20 years. Irritability sub-scores showed no differences except for higher scores in participants with 5–<10 if compared with those with >20 years to the predicted onset (Table 5).

## 4. Discussion

In this study, we analyzed the ENROLL-HD dataset with more than ten thousand participants with motor manifestations according to disease stages based on the TFC and potential influencing factors. We identified more than three thousand patients in disease stages 1 and 2 and more than two thousand in stage 3. Thus, the majority of ENROLL-HD participants were early-manifestation patients. Nevertheless, more than 800 participants were integrated as stage 4 and more than 300 as stage 5, which allowed group comparisons with regard to the functional, motor, neuropsychiatric, and cognitive manifestations. Additionally, we investigated more than five thousand pre-manifest participants with regard to their predicted onset and potential influencing factors on the TFC.

### 4.1. TFC as a Robust Criterion in Manifest HD: Quantifying Neurobiological Effects

In manifest HD, the TFC was significantly explained by the neurobiological factors of CAG-repeat length, age, and the interaction of both. We analyzed effects in one model, whereby the moderated regression analysis revealed that age, CAG repeats, and their interaction account for 20.9% of the TFC score´s variance—a significant part. Less distinct influences were observed for other factors, such as neuropsychiatric, educational, and cognitive influences. The hypothesis of age-related and CAG-dependent effects having a negative influence on the clinical course was postulated in an earlier approach of Rosenblatt et al., longitudinally assessing clinical data of *n* = 569 HD subjects, congruent with our findings in a large cohort [39,40]. This objectified classification of age- and genetic-dependent effects on functional impairments helps to assess to what extent interventional strategies or other genetic and epigenetic modifiers might improve disease-dependent dysfunctions. It helps furthermore to classify to what extent biomarkers are present other than age- and CAG-dependent findings [41,42]. Our analysis in the simple-slope model is even more relevant since earlier preclinical approaches in mice investigated the effects of the HD mutation on age-dependent pathways [43]. Additionally, a significant amount of an illustrated CAG impact on the TFC, which can be confirmed in our modeling approach, reveals that targeting CAG-repeat-dependent mechanisms as a therapeutic approach might explain a significant amount of beneficial effects on functional impairments [44]. Other influencing factors on the genetic cause of the disease, such as DNA repair mechanisms earlier investigated in HD, and their role in modulating the age of onset need to be discussed further [45,46].

The underlying neurobiological effect of age and CAG-repeat length on function is supported by a faster functional decline in subjects with higher CAGs and older patients in the simple slope analysis (Figure 2). This is presented here in one model based on a large clinical dataset. As a relevant factor and potential explanation of the faster decline in function and thus disease progression, one might assume somatic expansion as another relevant factor. Somatic expansion is known to increase with age and CAG-repeat length and has also been discussed to be responsible for a faster disease progression in juvenile or pediatric HD [40,47,48]. This might be a potential explanation for different progress in phenotypes with choreatic, akinetic-rigid, or hypokinetic symptoms [2].

### 4.2. Psychiatric and Cognitive Manifestation in Manifest HD and Its Influence on TFC-Based Stages

In our analyses, there was no independent significant impact of neuropsychiatric, cognitive, or educational aspects on disease stages according to the TFC (stages 1–5). Of course, there was a stage-dependent decline in cognition and behavioral changes with higher scores for depression in stages 2 and 3 and more irritability, psychosis, apathy, and less executive function (PBA-s) in higher stages as well as the highest scores for depression in stages 4, 5 and for irritability in stages 2 and 3 in the HADS-SIS.

All these changes, however, could not significantly explain the TFC-stage classification independently in manifest HD. Hence, our analysis does not support an influence of distinct cognitive and motor aspects on the TFC. Thus, we cannot confirm the independent effects of depression and apathy or cognitive impairments on the TFC as earlier described [29,30].

### 4.3. Analyzing Different “Pre-Manifesting Aspects” in Pre-Manifest HD

**Figure 3:**
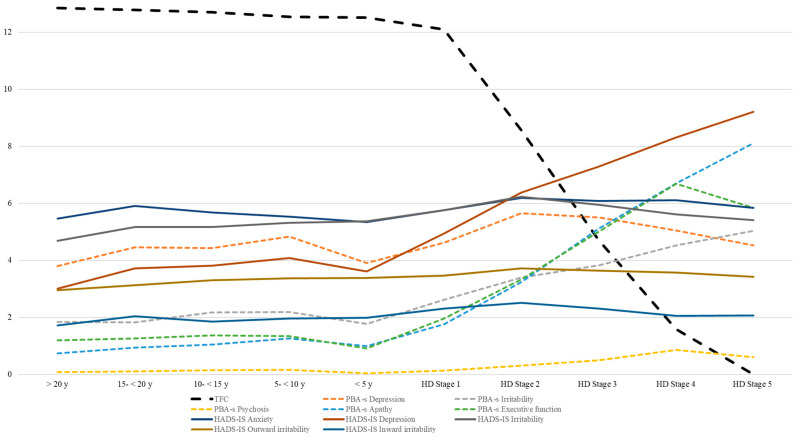
TFC and neuropsychiatric symptoms in HD according to stage.

**Figure 4:**
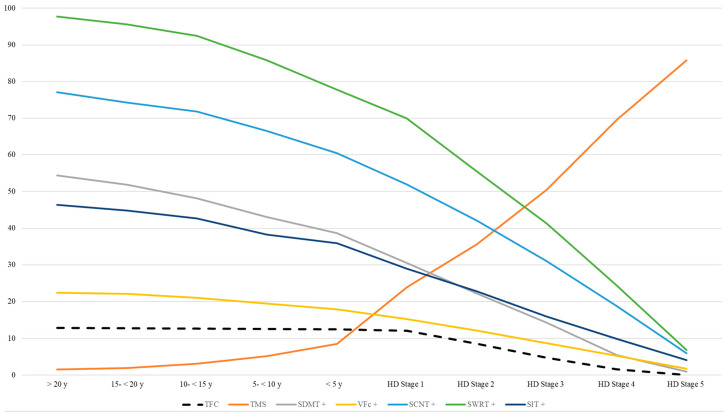
TFC, motor, and cognitive performance in HD according to stage. +: higher scores = better performance.

Analysis of neuropsychiatric changes in pre-manifest HD showed the highest impairments in those patients who were 5–15 years away from the predicted onset (Table 4 and Table 5). This is in line with earlier findings of psychiatric changes in prodromal HD [49]. As depicted in our illustrations (Figure 3 and Figure 4), there is a decline in cognitive test results and an increased rate of neuropsychiatric impairment in the prodromal stage of HD accompanied by a slight decline in TFC (<5 y up to <15 y to onset). However, these cognitive or neuropsychiatric aspects do not independently explain TFC changes. Thus, we confirm that the adequate use of the TFC score is limited to the manifest stages but less to the pre-manifest stage. Remarkably, the median data of the PBA-s revealed higher scores in the timeframe between 5 to <10, 10 to <15, and 15 to <20 than <5 years to the predicted onset, which confirms the hypothesis of psychiatric symptoms as frequently observed as early changes prior to the motor onset [1].

As a limitation, the prediction model and ENROLL-HD dataset revealed a comparatively low number of *n* = 138 pre-manifest participants in the <5 years to the predicted onset timeframe. This may be explained by the probability score, which is frequently used for the calculation of years to onset [16]. Nevertheless, this clinically orientated subdivision allows for a more precise assessment in pre-manifest HD [50]. As another limitation, neuropsychiatric symptoms were categorized as self-assessments without asking for other mental diseases, so, e.g., anxiety and depression could be misinterpreted. To minimize a potential bias, assessments by the clinical rater (PBA-s) were additionally analyzed.

Since these psychiatric and cognitive changes before the motor onset are difficult to interpret on an individual level and these changes are inconsistent (e.g., depression decreases in stage 5), it justifies the introduction of a biomarker stage in the pre-manifest stages as recently suggested by Tabrizi et al [51].

In summary, the moderated regression model and simple slope analysis appeared to be useful statistical approaches for analyzing the largest cohort of more than 10,000 motor- and pre-manifest HD participants according to influences on the TFC. We found that TFC was mainly explained by the neurobiological factors of CAG-repeat length and age. The TFC was confirmed as a robust measure of progression in manifest HD. There was some decline in cognitive test results and an increased rate of depression and apathy in the prodromal stage of HD, without independently influencing the TFC.

## Figures and Tables

**Table 1:** Demographic, motor, and functional data within HD stages: +: higher scores = better performance; #: higher scores = more impairment. Abbreviations: CAG: cytosine-adenine-guanine repeat length; ISCED, International Standard Classification of Education—educational level; CAP score: CAG-age product—index; HD: Huntington’s disease; TMS: Total Motor Score; TFC: Total Functional Capacity; y: years. SDMT, Symbol Digit Modalities test; VFc, verbal fluency test (category); SCNT, Stroop color naming test; SWRT, Stroop word reading test; SIT, Stroop interference test.

	HD Stage 1 *n =* 3319	HD Stage 2 *n =* 3580	HD Stage 3 *n =* 2296	HD Stage 4 *n* = 809	HD Stage 5 *n* = 310	*F*	*p*	*Part. Eta^2^*
Age (y); M (SD)	50.30 (11.95)	52.86 (12.49)	54.48 (12.82)	56.53 (12.85)	58.17 (13.95)	68.93	<0.001	0.026
CAG; M(SD)	43.73 (3.43)	43.92 (3.87)	44.40 (4.31)	44.69 (4.37)	45.63 (4.55)	33.27	<0.001	0.013
Sex (f/m) (%f)	1513/1806 (45.6)	1891/1689 (52.8)	1249/1047 (54.4)	465/344 (57.5)	183/127 (59.0)	75.27	<0.001	0.004
ISCED; M (SD)	3.67 (1.19)	3.43 (1.20)	3.21 (1.25)	2.97 (1.24)	2.84 (1.29)	93.97	<0.001	0.035
CAP-Score; M (SD)	475.53 (78.31)	507.03 (87.63)	548.37 (92.99)	587.65 (98.79)	632.34 (128.66)	523.96	<0.001	0.169
Disease duration (y); M (SD)	4.90 (6.33)	6.75 (5.62)	9.18 (6.69)	12.87 (8.76)	19.19 (14.45)	522.51	<0.001	0.169
HD Diagnosis (y); M (SD)	48.68 (12.09)	49.63 (12.75)	49.41 (13.22)	48.39 (13.77)	46.14 (13.17)	6.97	<0.001	0.003
Motor Onset (y); M (SD)	46.29 (11.79)	46.77 (12.27)	46.31 (12.78)	45.15 (13.43)	42.10 (12.94)	10.91	<0.001	0.004
TMS; M (SD) #	23.84 (11.31)	35.62 (14.00)	50.51 (17.10)	69.54 (17.33)	85.82 (16.24)	3056.04	<0.001	0.544
SDMT +	30.49 (11.01) (*n* = 3278)	22.19 (9.5) (*n* = 3470)	14.32 (8.91) (*n* = 1959)	5.45 (6.60) (*n* = 510)	0.97 (3.16) (*n* = 156)	1458.27	<0.001	0.384
VFc +	15.29 (5.20) (*n* = 3286)	12.12 (4.66) (*n* = 3537)	8.73 (4.31) (*n* = 2225)	5.32 (3.57) (*n* = 680)	1.77 (2.65) (*n* = 189)	124.97	<0.001	0.334
SCNT +	51.93 (14.10) (*n* = 3272)	42.02 (13.67) (*n* = 3512)	30.96 (13.37) (*n* = 2172)	18.82 (12.85) (*n* = 638)	5.94 (9.92) (*n* = 184)	1549.98	<0.001	0.388
SWRT +	69.91 (18.30) (*n* = 3281)	55.51 (17.61) (*n* = 3497)	41.27 (17.92) (*n* = 2140)	24.39 (17.44) (*n* = 626)	6.79 (12.31) (*n* = 181)	1682.56	<0.001	0.409
SIT +	28.92 (10.04) (*n* = 3038)	22.82 (9.70) (*n* = 3128)	15.96 (9.10) (*n* = 1721)	9.29 (7.76) (*n* = 423)	4.07 (6.65) (*n* = 74)	808.67	<0.001	0.279

**Table 2:** Comparison of neuropsychiatric data between different HD stages: #: higher scores = more impairment.

	HD Stage 1 *n* = 3319	HD Stage 2 *n* = 3580	HD Stage 3 *n* = 2296	HD Stage 4 *n* = 809	HD Stage 5 *n* = 310	*F*	*p*	*Part. Eta^2^*
Clinical rater (PBA-s)								
Depression; M (SD) #	4.63 (5.81) (*n* = 3315)	5.66 (6.65) (*n* = 3571)	5.51 (6.90) (*n* = 2278)	5.05 (6.30) (*n* = 775)	4.53 (5.77) (*n* = 205)	13.241	<0.001	0.005
Irritability; M (SD) #	2.62 (3.99)	3.40 (4.82)	3.83 (5.63)	4.53 (6.42)	5.04 (6.65)	42.44	<0.001	0.016
Psychosis; M (SD) #	0.14 (1.10)	0.31 (1.61)	0.50 (2.22)	0.86 (3.21)	0.61 (2.03)	31.74	<0.001	0.012
Apathy; M (SD) #	1.76 (3.03)	3.24 (3.93)	5.12 (4.96)	6.71 (5.86)	8.11 (6.47)	411.00	<0.001	0.139
Executive function; M (SD) #	1.97 (3.76)	3.33 (5.04)	5.01 (6.25)	6.69 (7.41)	5.84 (7.04)	199.82	<0.001	0.073
Self-report (HADS-IS)								
Anxiety; M (SD) #	5.76 (4.08) (*n* = 2055)	6.20 (4.31) (*n* = 2116)	6.09 (4.31) (*n* = 1093)	6.12 (4.34) (*n* = 269)	5.84 (4.54) (*n* = 46)	3.00	0.018	0.002
Depression; M (SD) #	4.95 (3.78)	6.38 (4.05)	7.30 (4.39)	8.32 (4.92)	9.22 (4.75)	93.28	<0.001	0.063
Irritability; M (SD) #	5.77 (4.39)	6.24 (4.76)	5.95 (4.73)	5.62 (4.62)	5.42 (4.01)	3.31	<0.001	0.002
Outward irritability; M (SD) #	3.47 (2.64)	3.73 (2.88)	3.65 (2.94)	3.58 (3.08)	3.43 (2.60)	2.45	0.044	0.002
Inward irritability; M (SD) #	2.31 (2.35)	2.51 (2.54)	2.31 (2.58)	2.06 (2.39)	2.07 (2.24)	3.30	0.011	0.002

**Table 3:** Significant group differences in post hoc Tukey-HSD tests for neuropsychiatric parameters in pairwise stage comparisons: #: higher scores = more impairment (stage comparisons without significant differences are not depicted).

Post Hoc Tukey-HSD	HD Stage Comparison		*p*
PBA-Depression #	Stage 1	Stage 2	<0.001
		Stage 3	<0.001
PBA-Irritability #	Stage 1	Stage 2	<0.001
		Stage 3	<0.001
		Stage 4	<0.001
		Stage 5	<0.001
	Stage 2	Stage 3	0.009
		Stage 4	<0.001
		Stage 5	<0.001
	Stage 3	Stage 4	<0.001
		Stage 5	<0.005
PBA-Psychosis #	Stage 1	Stage 2	<0.005
		Stage 3	<0.001
		Stage 4	<0.001
		Stage 5	<0.005
	Stage 2	Stage 3	<0.005
		Stage 4	<0.001
	Stage 3	Stage 4	<0.001
PBA-Apathy #	Stage 1	Stage 2	<0.001
		Stage 3	<0.001
		Stage 4	<0.001
		Stage 5	<0.001
	Stage 2	Stage 3	<0.001
		Stage 4	<0.001
		Stage 5	<0.001
	Stage 3	Stage 4	<0.001
		Stage 5	<0.001
	Stage 4	Stage 5	<0.001
PBA-Executive function #	Stage 1	Stage 2	<0.001
		Stage 3	<0.001
		Stage 4	<0.001
		Stage 5	<0.001
	Stage 2	Stage 3	<0.001
		Stage 4	<0.001
		Stage 5	<0.001
	Stage 3	Stage 4	<0.001
HADS-Anxiety #	Stage 1	Stage 2	<0.050
HADS-Depression #	Stage 1	Stage 2	<0.001
		Stage 3	<0.001
		Stage 4	<0.001
		Stage 5	<0.001
	Stage 2	Stage 3	<0.001
		Stage 4	<0.001
		Stage 5	<0.001
	Stage 3	Stage 4	<0.005
		Stage 5	<0.050
HADS-Irritability #	Stage 1	Stage 2	<0.050
HADS-Outward irritability #	Stage 1	Stage 2	<0.050
		Stage 4	<0.050

**Table 4:** Comparison of demographic, cognitive, and neuropsychiatric data in pre-manifest HD participants according to calculated years to onset: +: higher scores = better performance; #: higher scores = more impairment.

Years to Calculated Onset	<5 y *n* = 138	5–<10 y *n* = 1242	10–<15 y *n* = 1215	15–<20 y *n* = 920	>20 y *n* = 1634	*F*	*p*	*Part. Eta^2^*
Years to onset M (SD)	4.43 (0.47)	7.71 (1.40)	12.44 (1.43)	17.33 (1.43)	29.00 (8.60)	3938.84	<0.001	0.754
TFC; M (SD) +	12.51 (0.97)	12.54 (1.01)	12.70 (0.97)	12.78 (0.90)	12.85 (0.74)	22.01	<0.001	0.017
Age (y); M (SD)	44.61 (9.35)	46.78 (12.17)	41.54 (11.98)	32.22 (10.30)	33.76 (9.73)	267.16	<0.001	0.172
CAG; M(SD)	46.38 (2.94)	43.55 (2.95)	42.96 (2.73)	42.24 (2.11)	40.76 (1.94)	256.36	<0.001	0.217
Sex (f/m) (%f)	70/68 (50.7)	177/509 (59.0)	705/510 (58.0)	591/329 (64.2)	987/647 (60.4)	14.40	<0.010	0.006
ISCED	3.93 (1.16)	3.83 (1.16)	3.91 (1.13)	4.01 (1.09)	4.06 (1.08)	8.68	<0.001	0.007
CAP-Score; M (SD)	544.19 (55.02)	430.22 (32.56)	355.67 (18.36)	306.94 (16.73)	226.57 (46.43)	8164.42	<0.001	0.864
TMS; M (SD) #	8.52 (8.14)	5.19 (5.72)	3.12 (3.83)	1.92 (3.33)	1.53 (2.65)	212.15	<0.001	0.142
SDMT +	38.67 (11.40) (*n* = 135)	43.03 (11.79) (*n* = 1232)	48.08 (11.07) (*n* = 1204)	51.86 (10.96) (*n* = 917)	54.36 (10.83) (*n* = 1627)	226.91	<0.001	0.151
VFc +	17.86 (5.39) (*n* = 134)	19.44 (5.69) (*n* = 1235)	21.00 (5.51) (*n* = 1201)	22.08 (5.64) (*n* = 914)	22.38 (5.65) (*n* = 1625)	66.045	<0.001	0.049
SCNT +	60.49 (15.00) (*n* = 136)	66.50 (14.52) (*n* = 1230)	71.84 (14.21) (*n* = 1199)	74.29 (14.02) (*n* = 912)	77.09 (13.96) (*n* = 1625)	125.97	<0.001	0.090
SWRT +	77.78 (16.96) (*n* = 135)	85.84 (19.05) (*n* = 1232)	92.44 (17.14) (*n* = 1200)	95.59 (17.67) (*n* = 913)	97.76 (17.16) (*n* = 1626)	108.47	<0.001	0.078
SIT +	35.88 (11.18) (*n* = 128)	38.26 (10.92) (*n* = 1161)	42.64 (10.88) (*n* = 1133)	44.84 (10.65) (*n* = 856)	46.32 (10.68) (*n* = 1547)	113.06	<0.001	0.086
Clinical rater (PBA-s)								
Depression; M (SD) #	3.91 (5.90) (*n* = 137)	4.84 (6.40) (*n* = 1238)	4.44 (5.87) (*n* = 1213)	4.46 (5.85) (*n* = 917)	3.80 (5.27) (*n* = 1627)	6.148	<0.001	0.005
Irritability; M (SD) #	1.78 (3.65)	2.20 (4.03)	2.18 (3.75)	1.83 (3.25)	1.84 (3.25)	3.18	<0.050	0.002
Psychosis; M (SD) #	0.04 (0.38)	0.17 (1.51)	0.15 (1.05)	0.11 (0.88)	0.09 (0.78)	1.58	0.177	0.001
Apathy; M (SD) #	1.00 (2.28)	1.27 (2.74)	1.05 (2.47)	0.94 (2.39)	0.74 (1.96)	9.05	<0.001	0.007
Executive function; M (SD) #	0.92 (2.46)	1.35 (3.28)	1.37 3.18)	1.27 (3.25)	1.20 (3.00)	1.11	0.350	0.001
Self-report (HADS-IS)								
Anxiety; M (SD) #	5.35 (3.90) (*n* = 82)	5.54 (3.94) (*n* = 804)	5.68 (4.00) (*n* = 837)	5.91 (4.22) (*n* = 641)	5.47 (3.94) (*n* = 1179)	1.47	0.208	0.002
Depression; M (SD) #	3.61 (3.65)	4.09 (3.66)	3.82 (3.60)	3.72 (3.71)	3.01 (3.12)	13.48	<0.001	0.015
Irritability; M (SD) #	5.38 (4.11)	5.32 (4.15)	5.18 (4.02)	5.17 (4.10)	4.69 (3.88)	3.74	<0.010	0.004
Outward irritability; M (SD) #	3.39 (2.69)	3.37 (2.52)	3.31 (2.56)	3.13 (2.43)	2.96 (2.43)	4.17	<0.010	0.005
Inward irritability; M (SD) #	1.99 (2.02)	1.96 (2.20)	1.86 (2.06)	2.05 (2.35)	1.73 (2.05)	2.80	<0.050	0.003

**Table 5:** Significant group differences of post hoc Tukey-HSD tests in pre-manifest HD for neuropsychiatric parameters in pairwise comparisons: #: higher scores = more impairment (comparisons without significant differences are not depicted).

Post Hoc Tukey-HSD	Pre-Manifest HD Comparisons		*p*
	<5 y	15–<20 y	<0.050
		>20 y	<0.001
	5–<10 y	10–<15 y	<0.001
		15–<20 y	<0.001
		>20 y	<0.001
	10–<15 y	>20 y	<0.001
PBA-Depression #	5–<10 y	>20 y	<0.001
	10–<15 y	>20 y	<0.050
	15–<20 y	>20 y	<0.050
PBA-Apathy #	5–<10 y	15–<20 y	<0.050
		>20 y	<0.001
	10–<15 y	>20 y	<0.010
HADS-Depression #	5–<10 y	>20 y	<0.001
	10–<15 y	>20 y	<0.001
	15–<20 y	>20 y	<0.001
HADS-Irritability #	5–<10 y	>20 y	<0.010
HADS-Outward irritability #	5–<10 y	>20 y	<0.005
	10–<15 y	>20 y	<0.050
HADS-Inward irritability #	15–<20 y	>20 y	<0.050

## Data Availability

The data that support the findings of this study are available from the authors. Upon a reasonable request, the data can be provided with permission given by the Cure Huntington’s Disease Initiative (CHDI).
